# Robust molecular subgrouping and copy-number profiling of medulloblastoma from small amounts of archival tumour material using high-density DNA methylation arrays

**DOI:** 10.1007/s00401-013-1126-5

**Published:** 2013-05-14

**Authors:** Volker Hovestadt, Marc Remke, Marcel Kool, Torsten Pietsch, Paul A. Northcott, Roger Fischer, Florence M. G. Cavalli, Vijay Ramaswamy, Marc Zapatka, Guido Reifenberger, Stefan Rutkowski, Matthias Schick, Melanie Bewerunge-Hudler, Andrey Korshunov, Peter Lichter, Michael D. Taylor, Stefan M. Pfister, David T. W. Jones

**Affiliations:** 1 Division of Molecular Genetics, German Cancer Research Center (DKFZ), Im Neuenheimer Feld 280, 69120 Heidelberg, Germany; 2 Program in Developmental and Stem Cell Biology and The Arthur and Sonja Labatt Brain Tumour Research Centre, The Hospital for Sick Children, 555 University Avenue, Toronto, ON Canada; 3 Department of Laboratory Medicine and Pathobiology, University of Toronto, Toronto, ON Canada; 4 Division of Neurosurgery, The Hospital for Sick Children, 555 University Avenue, Toronto, ON Canada; 5 Division of Pediatric Neurooncology, German Cancer Research Center (DKFZ), Im Neuenheimer Feld 280, 69120 Heidelberg, Germany; 6 Department of Neuropathology, University of Bonn Medical Centre, Sigmund-Freud-Strasse 25, 53105 Bonn, Germany; 7 Genomics and Proteomics Core Facility, German Cancer Research Center (DKFZ), Im Neuenheimer Feld 280, 69120 Heidelberg, Germany; 8 Department of Neuropathology, Heinrich-Heine-University Düsseldorf, Moorenstrasse 5, 40225 Düsseldorf, Germany; 9 Department of Pediatric Hematology and Oncology, University Medical Center Hamburg-Eppendorf, Martinistrasse 52, 20246 Hamburg, Germany; 10 Department of Neuropathology, University of Heidelberg, Im Neuenheimer Feld 220, 69120 Heidelberg, Germany; 11 Clinical Cooperation Unit Neuropathology, German Cancer Research Center (DKFZ), Im Neuenheimer Feld 280, 69120 Heidelberg, Germany; 12 Department of Pediatric Oncology, Hematology and Immunology, Heidelberg University Hospital, Im Neuenheimer Feld 430, 69120 Heidelberg, Germany

It is now clear that medulloblastoma (MB), one of the most clinically challenging paediatric brain tumours, is not a single disease entity. Rather, it comprises four distinct molecular subgroups (Wnt pathway activated (WNT), Sonic hedgehog pathway activated (SHH), and the less well-characterised Group 3 and Group 4) [7, 15], which are highly divergent in terms of their patient demographics, underlying biology, and survival outcomes [4, 6]. These subgroups are becoming increasingly important, not only for refining the discovery of prognostic markers or therapeutic targets, but also for the design of prospective clinical trials. Patients with WNT subgroup tumours, for example, generally have a favourable prognosis, and may benefit from a reduction or omission of radiotherapy or chemotherapy to spare neurological side-effects or other toxicities, as is now being prospectively tested in upcoming trials both in North America and Europe. In contrast, patients with poor prognosis Group 3 tumours may benefit from intensification of up-front therapy. Furthermore, many new targeted therapeutics are likely to be efficacious in only one subgroup, such as smoothened inhibitors for SHH pathway-driven MB [1, 2]. A phase III clinical trial randomising SMO inhibition against standard of care in relapsed SHH-MB patients will start recruiting in mid-late 2013. A method for accurate and robust classification into tumour subgroups that is applicable to standard pathology specimens is therefore of key clinical relevance.

The MB subgroups were originally defined based on gene expression profiling from fresh-frozen tumour material [7]. Whilst there are methods to apply such an RNA-based analysis to formalin-fixed paraffin-embedded (FFPE) material, classification accuracy is inferior to that obtained with frozen tissue, particularly when analysing older samples [9]. Furthermore, the use of immunohistochemistry as an alternative subgrouping method [7] has proved difficult to standardise across multiple neuropathology laboratories. The use of a DNA-based platform for subgrouping has clear advantages due to the superior stability of DNA compared with RNA. Methylation profiling has recently been applied for the subgrouping of large series of, for example, glioblastoma and chronic lymphocytic leukaemia samples [5, 10, 14]. It has also been proposed as being suitable for medulloblastoma subclassification, although the older Illumina GoldenGate platform assessed only a limited subset of genes, and a proportion of samples remained unclassifiable [12]. Also, whilst the concordance between methylation and expression reported by Schwalbe et al. was fairly good (81.5 %), some WNT and SHH-subgroup tumours were misclassified—a clinically important distinction for forthcoming trials. We therefore applied the Illumina Infinium HumanMethylation450 BeadChip array (450k array) to generate genome-wide methylation profiles of a large series of medulloblastoma samples (see Supplementary Methods). The first cohort comprised 107 frozen MB samples collected within the ICGC PedBrain Tumor Project (Heidelberg cohort) [3]. Of these, 86 had matching Affymetrix U133 plus 2.0 expression array data, allowing for a direct comparison between the subgroup classifications of the two methods. Unsupervised *k*-means consensus clustering on all CpG probes with a standard deviation >0.25 (*n* = 21,092) clearly indicated the presence of four subgroups (Fig. 1a). These methylation subgroups very closely recapitulated the gene expression subgroups of the matching tumours (95.3 % concordance, Rand index = 0.86, *p* < 0.0001; Fig. 1b, c). As expected from previous subgrouping studies, the discordant cases involved switches between Group 3 and Group 4, while the WNT and SHH groups were clearly distinct (Fig. 1c, d).

**Fig. 1:**
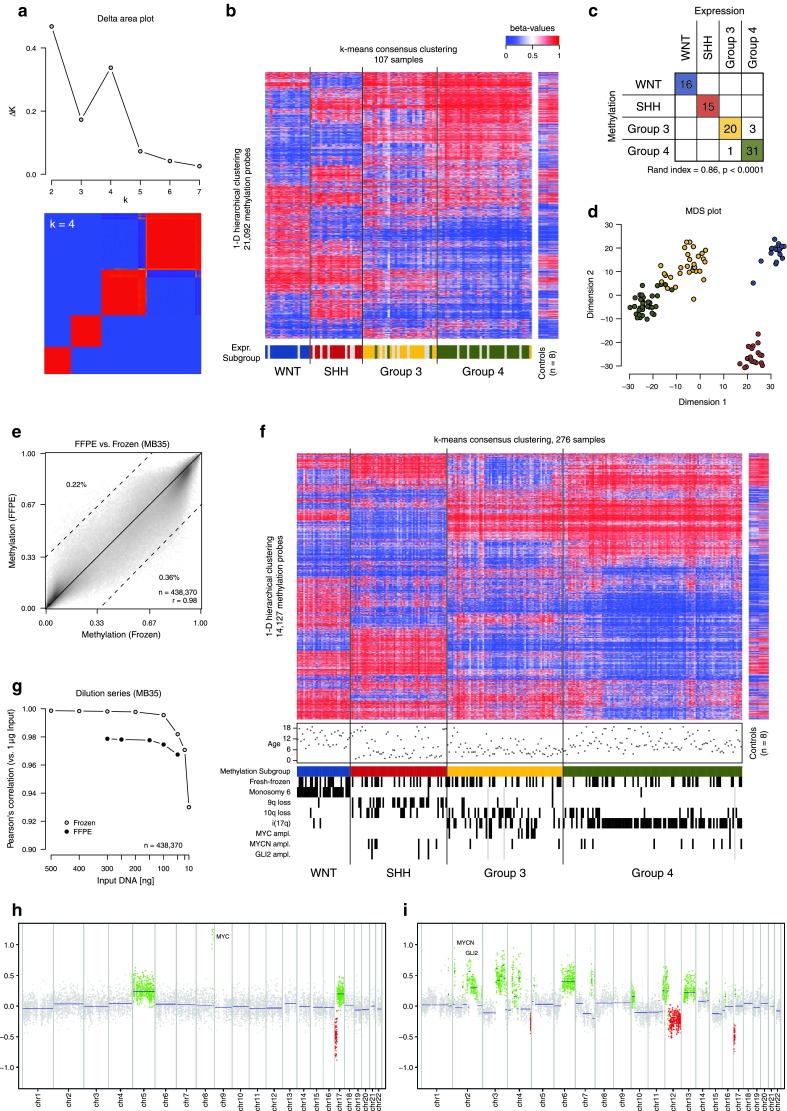
**a** *k*-means consensus clustering of the Heidelberg fresh-frozen cohort (*n* = 107) using the 21,092 most variable CpG probes (SD > 0.25) indicates the presence of four major subgroups in the DNA methylation data. **b** Heatmap of DNA methylation values within the four subgroups derived from the consensus clustering. The gene expression subgroup of the matched samples is indicated below the heatmap. Eight normal cerebellum controls are also shown for comparison. **c** Concordance chart of the gene expression versus DNA methylation-derived subgroups for each sample. **d** Multidimensional scaling (MDS) analysis of the same samples and same CpG probes used for the consensus clustering. **e** Correlation of DNA methylation values derived from fresh-frozen and FFPE material from a single tumour sample. **f** Heatmap of DNA methylation values across a combined set of the Heidelberg fresh-frozen cohort and the FFPE tumour cohort (*n* = 276). Patient age and copy-number events derived from the 450k array data are indicated below the heatmap. **g** Correlation of DNA methylation values from a dilution series of fresh-frozen and FFPE tumour DNA from a single sample. **h** Copy-number plot of a Group 3 medulloblastoma from the FFPE series, showing stereotypic *MYC* amplification and i(17q). **i** Copy-number plot of an SHH medulloblastoma from the FFPE series displaying evidence of dramatic structural changes, reminiscent of chromothripsis

The 450k array is also suitable for analysis of DNA from FFPE material. Profiling of the same tumour from both frozen and FFPE material (*n* = 3) showed a higher correlation than the maximum correlation between different donors, indicating the robustness of this assay for archival tissue (mean Pearson’s correlation for paired samples, *r* = 0.987, maximum correlation for any two unpaired samples, *r* = 0.975; Fig. 1e). We therefore profiled an independent set of 169 FFPE MB samples using the methylation array. These were confirmed as unique samples using SNP genotyping probes from the array platform, which allows for testing to detect duplicate samples from the same patient. Consensus clustering of these samples together with the fresh-frozen cohort did not show any grouping by type of material, and all samples could be assigned a subgroup annotation (Fig. 1f). Using a reduced 48–CpG signature to train a support vector machine (SVM) classifier on the frozen tissue cohort, we were able to predict a tumour subgroup for the FFPE samples with an extremely close match to the clustering subgroup (97.6 % concordance, Rand index = 0.93, *p* < 0.0001; Supplementary Figure 1a, b). This signature allows for simple classification of single clinical samples without the need for comparison against a larger reference dataset. The data for these signature probes for the Heidelberg frozen tumour cohort are given in Supplementary Table 1.

We also investigated the impact of input DNA quantity on resulting data quality, using a dilution series of a single sample down to as little as 10 ng input material (the manufacturer’s recommended input is 500 ng for fresh-frozen material or 250 ng for FFPE DNA). For both fresh-frozen and FFPE DNA, there was a very high correlation between profiles at all input quantities down to 50 ng (Fig. 1g). The frozen sample was also tested with 25 and 10 ng of input, resulting in a slightly lower correlation. However, even at 10 ng, the sample would still have been accurately classified as an SHH tumour (Supplementary Figure 1c). Thus, this platform may be suitable for molecular subgrouping even when DNA quantities are limiting.

To further validate the broad applicability of this technique, we examined an additional independent tumour cohort with matching expression subgrouping data (derived as previously described [8]), for which the 450k arrays were run in an entirely separate facility (Toronto cohort, *n* = 60). SNP genotyping from the array indicated that four samples were also part of the Heidelberg frozen cohort. As with the frozen versus FFPE comparison, the correlation between these paired samples run in different facilities was higher than any other pairwise comparison (mean Pearson’s correlation for paired samples, *r* = 0.988). The derived methylation subgroups of the remaining 56 unique samples again gave a very close overlap with the expression-defined subgroups (94.6 % concordance, Rand index = 0.86, *p* < 0.0001), showing the robustness of this platform for the classification of MB, independent of where the data are generated (Supplementary Figure 2a, b).

A further major benefit of using this comprehensive array platform rather than a targeted gene panel is the ability to generate genome-wide copy-number profiles using the intensity measures of the methylation probes, with a good concordance to other copy-number platforms such as CGH or SNP arrays, as we have recently described [14]. This allowed us to detect clinically relevant copy-number aberrations, such as *MYC/MYCN/GLI2* gene amplifications, from the FFPE as well as the frozen tumour samples (Fig. 1f, h). Stereotypic MB copy-number changes showed the expected subgroup distribution (e.g. monosomy 6 in WNT tumours, 9q/10q loss in SHH, *MYC* amplification in Group 3, i(17q) in Group 3/Group 4; Fig. 1f). For 66 samples from the Heidelberg cohort, copy-number data from whole-genome sequencing (WGS) were also available, and were assessed for the alterations indicated in Fig. 1f. All scoring was consistent between WGS and 450k array profiles. Furthermore, 10/60 SHH-MBs showed patterns of dramatic copy-number change, reminiscent of chromothripsis [13] (Fig. 1i). We have previously linked this phenomenon to *TP53* mutations (typically germline) in SHH-MB [11]. This tool may therefore aid in identifying medulloblastoma patients with a particularly high risk of having underlying Li Fraumeni syndrome.

In summary, we demonstrate here a method for reliable classification of medulloblastoma into molecular subgroups, and tumour copy-number profiling, using a commercially available DNA methylation array platform that performs well on either frozen or FFPE tumour material. We also show that this technology can be reproducibly applied with low amounts of starting material, at different institutes, and with the benefit of easier handling compared with FFPE-derived RNA. We therefore believe that this platform holds great potential for refining the information obtainable from large, archival tumour series. Most importantly, we also expect that this will become one of the key technologies for risk stratification and patient cohort selection in the next generation of large, biology-led, multi-centre clinical trials.

## Electronic supplementary material

Below is the link to the electronic supplementary material.

Supplementary material 1 (PDF 2,969 kb)

Supplementary material 2 (XLS 123 kb)
