# Supplementary material for: Robust molecular subgrouping and copy-number profiling of medulloblastoma from small amounts of archival tumour material using high-density DNA methylation arrays
Source: Acta Neuropathol. 2013 May 14;125(6):913–6. doi: 10.1007/s00401-013-1126-5 (PMC3661908; doi:10.1007/s00401-013-1126-5)
Supplement: Supplementary file 1 — Supplementary material 1 (PDF 2,969 kb) [file 401_2013_1126_MOESM1_ESM.pdf]

DNA Methylation Array Data Generation

All tumour material was collected in accordance with research ethics board approval at the contributing centres. Four adult and four foetal cerebellar control samples were obtained from a commercial source (BioChain, USA). DNA methylation profiling using the Illumina Infinium HumanMethylation450 BeadChip array was performed according to the manufacturer's instructions at the DKFZ Genomics and Proteomics Core Facility (Heidelberg, Germany). Unless indicated differently, >500 ng of DNA was used as input material for fresh-frozen samples. 250 ng of DNA was used for most formalin-fixed paraffin-embedded (FFPE) tissues, or less if amounts were limited (>100 ng in all cases). Basic array processing was performed using Illumina GenomeStudio V2011.1 (Methylation Module version 1.9.0, content descriptor version 1.2). Signal intensities were obtained without background subtraction and normalised to internal controls. Samples from the Toronto cohort were processed using identical parameters (R package: minfi). beta-values were used for downstream methylation analyses and no further normalisation or transformation steps were performed. All samples were checked for duplicates by pairwise correlation of the 65 genotyping probes on the 450k array.

Expression Array Data Generation

Gene expression profiling was performed on the Affymetrix GeneChip Human Genome U133 Plus 2.0 array at the Microarray Department of the University of Amsterdam (Netherlands). Sample library preparation, hybridisation, and quality control were performed according to protocols recommended by the manufacturer. The MAS5.0 algorithm of the GCOS program (Affymetrix Inc) was used for normalisation of the expression data. Quality of the arrays was ensured by inspection of the beta-actin and GAPDH 5′-3′ ratios as well as the percentage of present calls generated by MAS5.0. Expression values were $\log_2$-transformed and subsequently normalised by *z*-score transformation. Probes mapping to the X and Y chromosomes were excluded.

Data Processing

Downstream analyses were performed in R [1]. The following criteria were applied to filter the data: removal of probes targeting the X and Y chromosomes (n = 11,551), removal of probes containing a single-nucleotide polymorphism (dbSNP132 Common) within five base pairs of and including the targeted CpG-site (n = 24,536), and probes not mapping uniquely to the human reference genome (hg19) allowing for one mismatch (n = 9,993). In total, 438,370 probes were kept for analysis. Missing values were imputed using the K Nearest Neighbor algorithm and default settings (R package: impute) [2].

For unsupervised consensus clustering of the fresh-frozen cohort we selected the 21,092 most variably methylated probes (standard deviation >0.25) across the dataset. The consensus matrix was calculated using the *k*-means algorithm (10 random starting sets, maximum of 1,000 iterations) on a fraction of probes (0.7) in 1,000 iterations for k = 2 to 7 (R package: clusterCons) [3, 4]. We applied hierarchical clustering (euclidean distance, average linkage) for reordering of the methylation probes in the heatmap plots (y-axis). Euclidean distance was also used for multidimensional scaling (MDS) analysis (R function: cmdscale). The p-value associated with the Rand index was calculated by performing a permutation test using 100,000 iterations.

Similar consensus clustering was performed for the combined Heidelberg series (fresh-frozen and FFPE, n = 14,127 probes, standard deviation >0.25) and the gene expression data (n = 2,212 probes, variance >2.5).

Technical comparisons

For the comparison of fresh-frozen and FFPE input material, dilution series and samples analysed at different institutes, the Pearson product-moment correlation coefficient was calculated as a measure of technical variability. All 438,370 probes that passed the filtering steps described above were used. In the dilution series, samples were compared to a fresh-frozen sample using 1 μg of DNA as input material.

Support Vector Machine class prediction

The reduced 48-CpG signature was derived from the fresh-frozen cohort using the same 21,092 probes as for the consensus clustering (standard deviation >0.25). For each subgroup, the most significantly differentially methylated probes were determined by comparing one subgroup against all others, including the normal cerebellum control samples (Wilcoxon rank-sum test). Probes were ranked by increasing p value and decreasing difference in mean methylation. The most highly ranked probes were included in the signature (6 for WNT and SHH, 18 for Group 3 and Group 4 subgroups), omitting probes located within 1Mb of an already included, higher ranked probe. A support vector machine (SVM) was trained on the fresh-frozen cohort (R package: e1071, parameters: type="C-classification", kernel="linear", scale=FALSE) and applied to predict medulloblastoma subgroups from the fresh-frozen, FFPE and Toronto cohort, as well as the fresh-frozen and FFPE dilution series.

Copy-number profiling

Low-resolution copy-number variations were detected from the 450k array as previously described [5]: In a first step, the signal intensities of both methylated and unmethylated signals were combined. Probes found to be highly variant in eight normal cerebellum samples were excluded from the analysis according to the following criteria: Removal of probes not within the 0.05 and 0.85 quantile of median summed values or over the 0.8 quantile of the median absolute deviation. Log-ratios of samples to the median value of control samples were calculated, and sample noisiness was determined as the median absolute deviation of adjacent probes. Probes were then combined by joining 20 adjacent probes, and resulting genomic windows less than 100 kb in size were iteratively merged with adjacent windows of smaller size to reach a minimum of 100 kb. Windows of more than 5 Mb were excluded from analysis, resulting in a total of 8,681 windows throughout the genome. For each window, the median probe value was calculated and shifted to minimize the median absolute deviation from all windows to zero for every sample. Segmentation was performed by applying the circular binary algorithm (CBS, R package: DNAcopy [6]) using the following settings: min.width=10, nperm=32000, alpha=0.001, undo.splits="sdundo", undo.SD=2.2. The median value of windows contained in each segment was calculated, and classified as homozygous or hemizygous deletion, neutral, gain or high-level amplification by the following manually-defined thresholds: -0.8, -0.2, 0.1 and 0.8. For all copy-number changes described in the main text or figures, automatic scoring was verified by manual curation of the respective loci for each individual profile.

1. R Development Core Team. *R: A language and environment for statistical computing*, (R Foundation for Statistical Computing, Vienna, Austria, 2010).
2. Troyanskaya, O. *et al.* Missing value estimation methods for DNA microarrays. *Bioinformatics* **17**, 520-5 (2001).
3. Monti, S., Tamayo, P., Mesirov, J. & Golub, T. Consensus clustering: A resampling-based method for class discovery and visualization of gene expression microarray data. *Machine Learning* **52**, 91-118 (2003).
4. Wilkerson, M.D. & Hayes, D.N. ConsensusClusterPlus: a class discovery tool with confidence assessments and item tracking. *Bioinformatics* **26**, 1572-1573 (2010).
5. Sturm, D. *et al.* Hotspot mutations in H3F3A and IDH1 define distinct epigenetic and biological subgroups of glioblastoma. *Cancer Cell* **22**, 425-37 (2012).
6. Olshen, A.B., Venkatraman, E.S., Lucito, R. & Wigler, M. Circular binary segmentation for the analysis of array-based DNA copy number data. *Biostatistics* **5**, 557-572 (2004).

Supplementary Figure 1

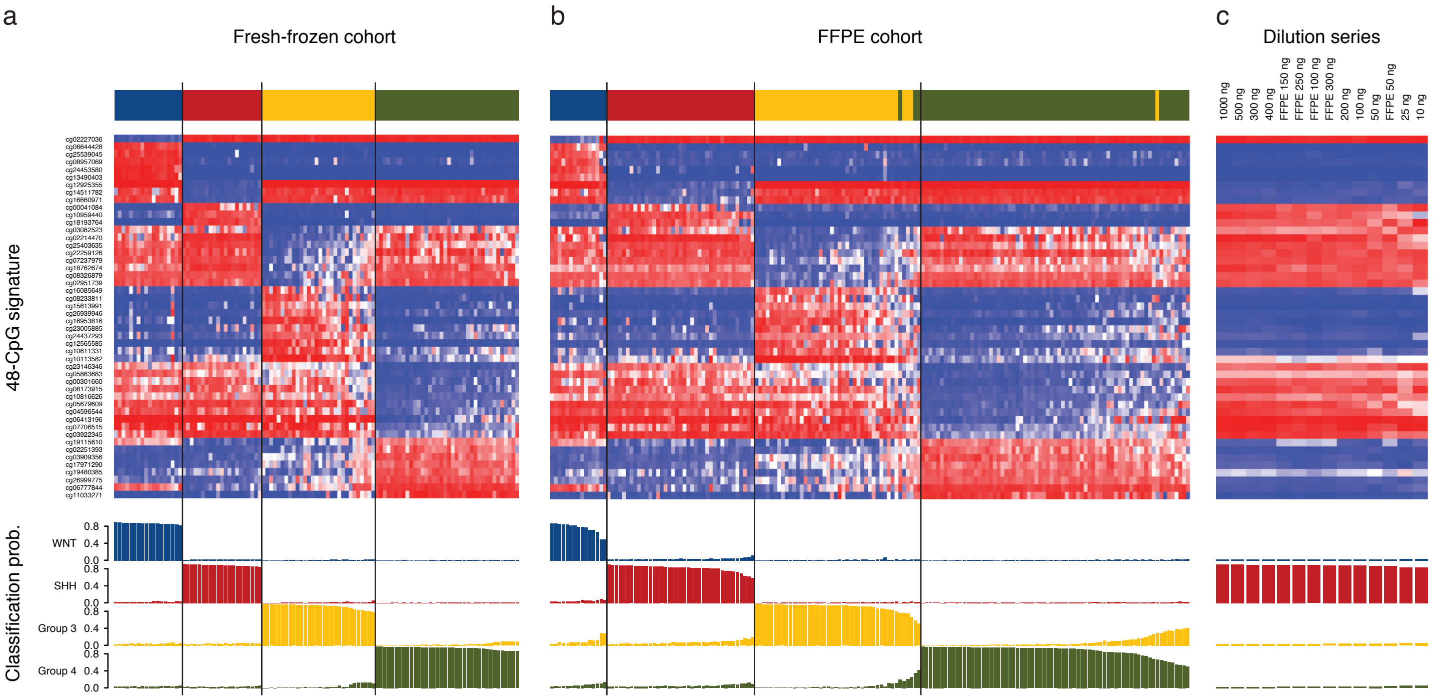

Figure Legend

a Heatmap of methylation values for the fresh-frozen tumour cohort (n = 107) for the 48-CpG probes from the support vector machine (SVM) subgroup classifier. The SVM classifier was trained on this tumor cohort. Samples are sorted per-subgroup according to their associated classification probability, as indicated below the heatmap.

b Heatmap of methylation values for the FFPE tumour cohort (n = 169) based on the SVM classifier depicted in (a). Methylation subgroup assignments from consensus clustering are indicated above the plot. Samples are sorted per-subgroup according to their associated classification probability, as indicated below the heatmap.

c Heatmap of methylation values for individual arrays from the fresh-frozen tissue and FFPE dilution series based on the SVM classifier depicted in (a). Samples are sorted according to their associated classification probability, as indicated below the heatmap. All samples would be correctly classified as SHH subgroup with high probability.

Supplementary Figure 2

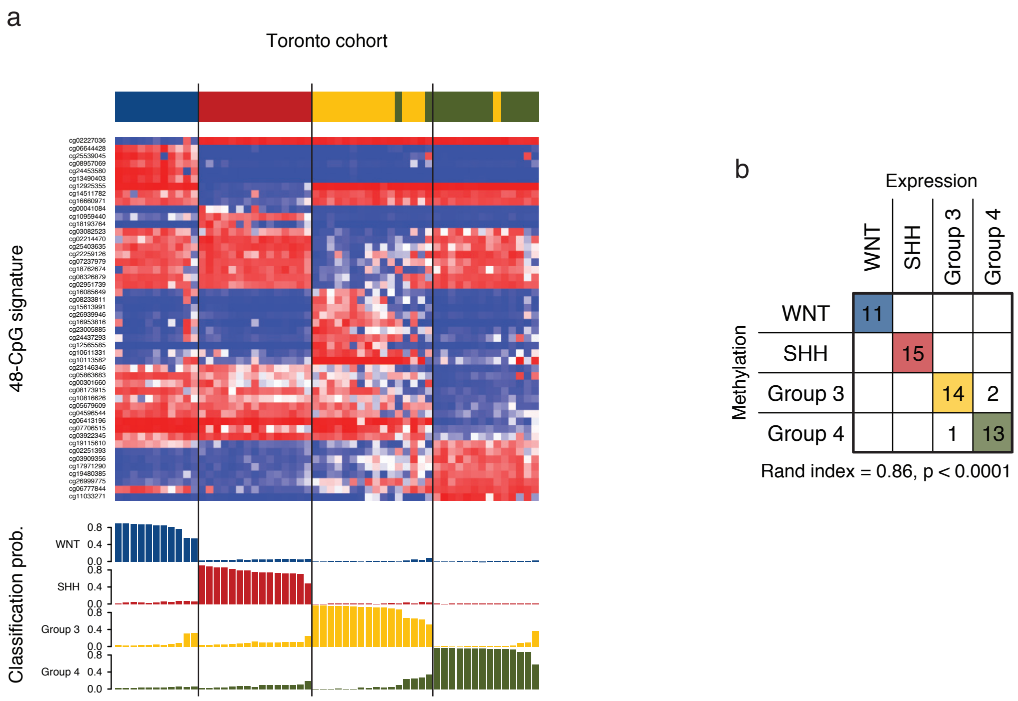

Figure Legend

a Heatmap of methylation values for the Toronto tumour cohort (*n* = 56) based on the SVM classifier depicted in Supplementary Figure 1a. Subgroup assignments from gene expression profiling are indicated above the plot. Samples are sorted per-subgroup according to their associated classification probability, as indicated below the heatmap.

b Concordance chart of the gene expression versus DNA methylation-derived subgroups for each sample.
